# Erythematous variant of inverse pityriasis versicolor in skin of colour

**DOI:** 10.1093/skinhd/vzae011

**Published:** 2025-01-20

**Authors:** Shreya Deoghare, Ajay Dodeja

**Affiliations:** Department of Dermatology, Venereology and Leprosy, N.K.P. Salve Institute of Medical Sciences and Research Centre & Lata Mangeshkar Hospital, Nagpur, India; Department of Dermatology, Venereology and Leprosy, N.K.P. Salve Institute of Medical Sciences and Research Centre & Lata Mangeshkar Hospital, Nagpur, India

A 30-year-old man (Fitzpatrick skin type IV) presented with asymptomatic, multiple well-defined, round to oval erythematous nonblanchable macules, with scaling on the left cubital fossa, for a duration of 1 month (Figure 1). There was no involvement of other body parts, and he denied use of topical irritants or perfumes, history of other systemic symptoms, drug intake, recent illness, or other significant past medical or family history. The lesion was earlier diagnosed as irritant contact dermatitis, and did not respond to mometasone furoate 0.1% cream, which was to be applied twice daily, as per the prescription.

**Figure 1:**
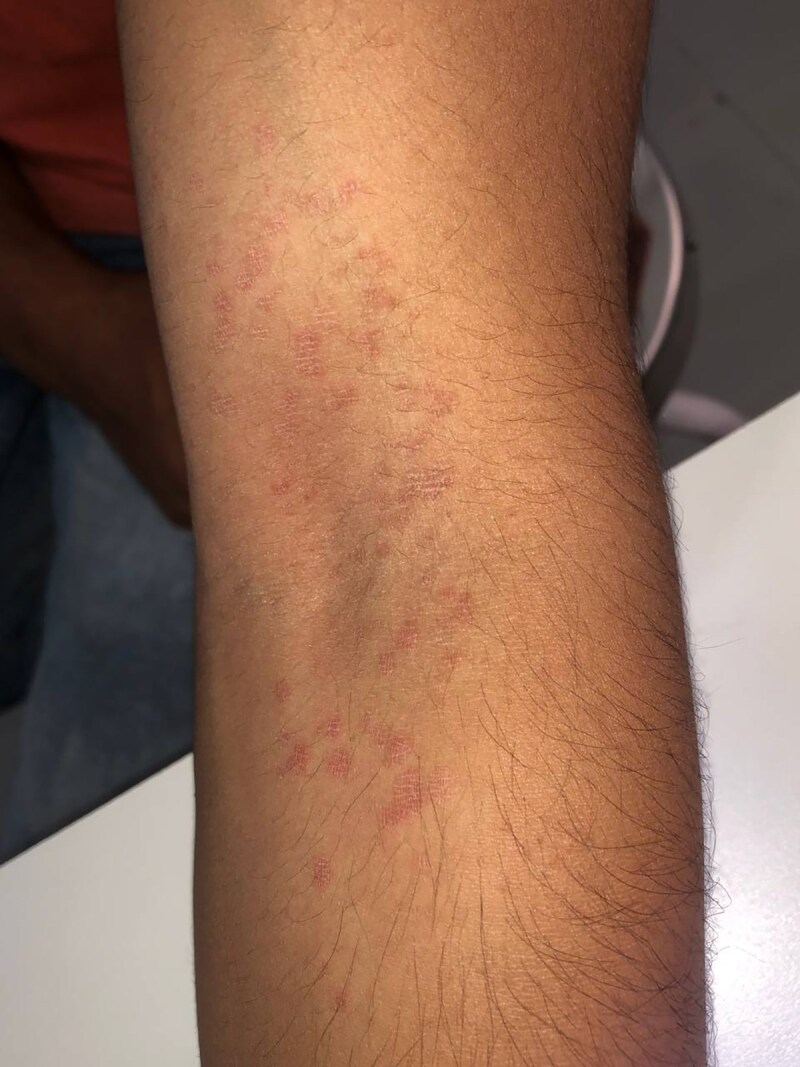
Multiple well-defined, round to oval erythematous nonblanchable macules, with scaling on left cubital fossa.

Dermoscopic examination (polarized light, Dermlite DL4) revealed fine scaling along skin creases, giving the appearance of a fish-net or wire fence pattern,^1^ on an erythematous background. Scaling was absent between the skin creases.^1^ A microscopic evaluation of scales soaked in potassium hydroxide (KOH) demonstrated the typical spaghetti and meatball appearance of the hyphae and spores, respectively (Figure 2).^2^ Based on this, a final diagnosis of the erythematous variant of pityriasis versicolor was made (Figure 3). He was prescribed 2% ketoconazole cream to be applied locally twice daily for 2 weeks, and advised on measures to prevent recurrence, like maintaining skin dryness and prophylactic use of antifungal body wash.

**Figure 2:**
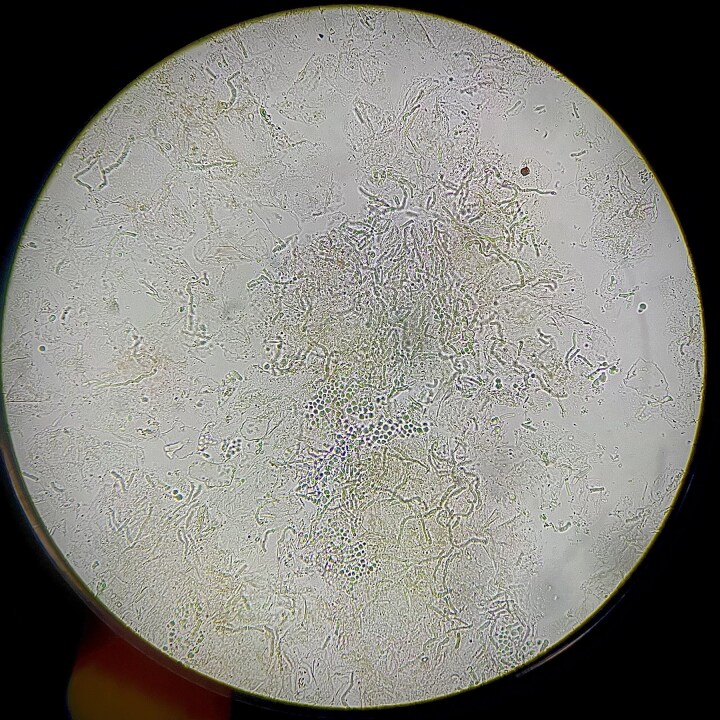
Potassium hydroxide (KOH) mount of the scales, demonstrating spaghetti and meatball appearance of the hyphae and spores, respectively.

**Figure 3:**
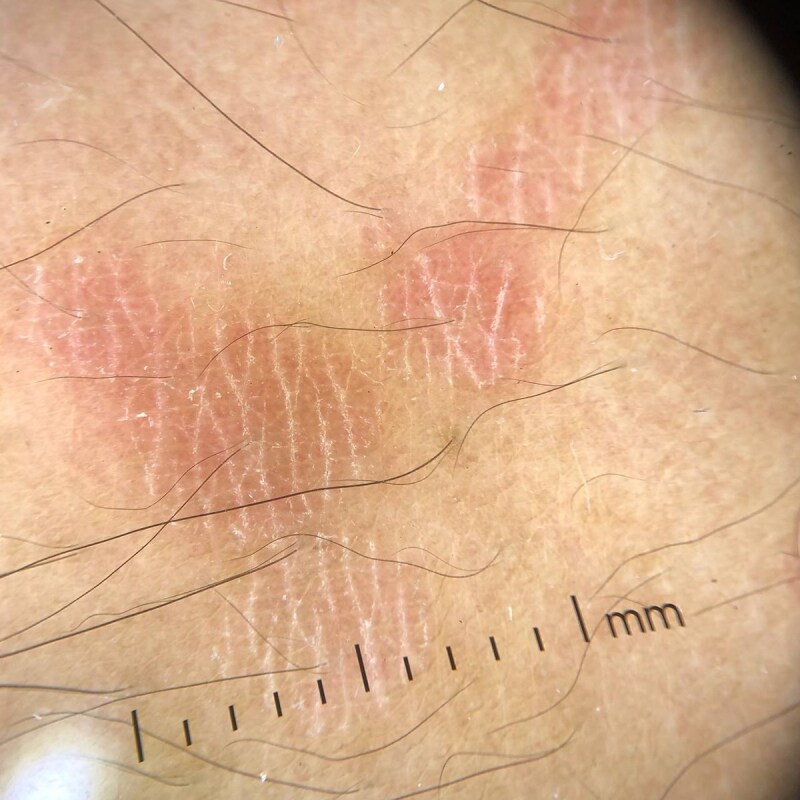
Dermoscopic examination (polarized light, Dermlite DL4) showing fine scaling along skin creases, on an erythematous background. Scaling was absent between the skin creases.

Pityriasis versicolor or tinea versicolor, caused by *Malassezia furfur*, presents in sebaceous regions such as the scalp, face, chest and upper back, and is more prevalent among adolescents and young adults due to heightened sebum production.^3^ It can present with ‘versi’ or varied color and morphological presentations, including hypopigmentation, hyperpigmentation, erythematous, papular, folliculocentric and atrophic variants.^3^ Hypopigmentation, mostly in darker skin tones, results from azelaic acid inhibiting melanocyte tyrosinase activity, while hyperpigmentation, common in lighter skin tones, is due to a hyperaemic inflammatory response and the formation of large melanosomes.^3^ Additionally, the fungus induces delicate scaling by enzymatically loosening the horny layer.^3^

The uniqueness of our case lies in a patient with a darker skin tone presenting with the erythematous variant of pityriasis versicolor at the uncommon location of the cubital fossa. The dermoscopic features in our case were in line with existing literature;^1^ this helped us to diagnose the inverse form of pityriasis versicolor, or tinea inversicolor, distinguishing it from other common dermatoses of that area, such as tinea corporis and irritant contact dermatitis. The erythematous appearance likely results from an inflammatory response insufficient to cause hyperpigmentation. This case underscores the value of dermoscopy in clinical practice and highlights the rare presentation of pityriasis versicolor in darker skin types, making it a compelling read for dermatologists.

## Data Availability

The data underlying this article will be shared on reasonable request to the corresponding author.

## References

[1] Thomas N, Malakar S. Dermoscopy: an easy way to solve the diagnostic puzzle in pityriasis versicolor. Indian J Dermatol Venereol Leprol 2019; 85:664. 30117461 10.4103/ijdvl.IJDVL_816_16

[2] Karray M, McKinney WP. Tinea versicolor. In: *StatPearls* [Internet]. Treasure Island (FL): StatPearls Publishing, 2024. Available at: https://www.ncbi.nlm.nih.gov/books/NBK482500/ (last accessed July 2024). 29494106

[3] Leung AK, Barankin B, Lam JM et al Tinea versicolor: an updated review. Drugs Context 2022; 11:2022-9-2. 10.7573/dic.2022-9-2 PMC9677953 36452877

